# Innovations in treatment of psychosis in southeast europe

**DOI:** 10.1192/j.eurpsy.2023.193

**Published:** 2023-07-19

**Authors:** N. Jovanovic

**Affiliations:** Queen Mary University of London, London, United Kingdom

## Abstract

Southeast Europe (SEE) is home to 70 million people living across 12 countries. These countries share similar socioeconomic background and tradition of healthcare systems, although many differences exist in the organisation of mental health care. The region has been called ‘the blind spot on the global mental health map’ due to lack of research and innovations in mental health care. In recent years European Commission has invested significant resources to improve mental health care of individuals with severe mental disorders in Southeast Europe. Two major, recently completed, projects are Recover-e and Impulse. This presentation will focus on findings from the Impulse study. We will start with an overview of evidence-based, non-pharmacological interventions for individuals with psychosis, with focus on what is offered and implemented in 12 SEE countries. Next, we will present key findings from the hybrid type II effectiveness-implementation psychosocial randomised-controlled trial. We will show an evidence-based approach how to improve psychosocial aspect of treatment of individuals with psychotic disorders. Mental health services that offer a combined-therapy approach, including psychosocial interventions and pharmacotherapy, can ensure holistic care, preferred by patients and their families. Discussion will include the role of implementation science tool in mental health care and sustainability of implemented interventions.

**Disclosure of Interest:**

None Declared

